# A Delayed Case of Pericarditis Following Recovery From COVID-19 Infection

**DOI:** 10.7759/cureus.14397

**Published:** 2021-04-10

**Authors:** Ann Kaminski, Michael Albus, Michael Mohseni, Haares Mirzan, Michael F Harrison

**Affiliations:** 1 Emergency Medicine, Mayo Clinic, Jacksonville, USA; 2 Internal Medicine, Mayo Clinic, Jacksonville, USA

**Keywords:** covid-19, corona virus disease, ultrasound (u/s), pericardial diseases, covid pericarditis, pericardial effusion

## Abstract

Pericarditis is a rare cardiac complication of coronavirus 19 (COVID-19) infection. Recent case reports describe severe sequelae of pericarditis, including cardiac tamponade, developing within days of initial COVID-19 symptoms. We present a case of pericarditis with slower onset and milder symptoms, developing over a period of a few weeks in an immunocompetent male who recovered from COVID-19 several months earlier.

A 65-year-old male presented to an emergency department several times for one week of worsening chest and neck symptoms, along with fever. He had been symptom-free after a three-day course of cough, myalgias, and fever with positive COVID-19 testing, approximately 70 days earlier. He was ultimately admitted for fever and pericarditis with an associated pericardial effusion and positive PCR testing for COVID-19.

Pericarditis should be considered in the differential diagnosis for patients with COVID-19 and unexplained persistent chest symptoms. The possibility of recurrent or atypical latent infection should additionally be considered in the months following the initial COVID-19 infection. Bedside ultrasound may facilitate early diagnosis and management of COVID-19 associated pericarditis.

## Introduction

The first case of severe acute respiratory syndrome coronavirus (SARS-CoV-2), commonly referred to as coronavirus 19 (COVID-19), was diagnosed in China in late 2019 [1]. Since that time, COVID-19 has become a global pandemic with the number of confirmed cases heading towards 100 million [2,3]. The mortality rates associated with COVID-19 infection are difficult to calculate with a high degree of accuracy due to uncertainty about the magnitude of the denominator [4,5]. However, these rates are estimated to range from 0.1% to 17.5% [4,5]. In addition to mortality, significant population morbidity has been attributed to COVID-19 infection in recovered cases [6]. COVID-19 sequelae include pulmonary complications such as parenchymal lung fibrosis [6], hemorrhagic and ischemic cerebrovascular complications [7], hematological complications including hypercoagulability and lymphopenia [8], and cardiac complications including cardiomyopathy and arrhythmia [9,10]. While some of these complications may persist chronically after recovery from the acute infection, the majority of these complications do initially present during the acute and sub-acute infectious period. Late complications associated with recovery from COVID-19 infection can include memory impairments and chronic fatigue, as part of a spectrum of chronic disease in a population of recovered COVID-19 patients described as “long haulers” [11].

Pericarditis is a rare extrapulmonary complication of COVID-19 infection and has been described in only a handful of cases [12-14]. However, these cases describe pericarditis diagnosed within one week of the initial diagnosis of COVID-19 infection and with emergent intervention required to stabilize tamponade physiology. In contrast, we present a case of acute pericarditis with mild initial symptoms as a late complication following recovery from a mild case of COVID-19 versus reinfection in an immunocompetent adult male.

## Case presentation

A 65-year-old male with a past medical history of obstructive sleep apnea, hypothyroidism, and atrial fibrillation presented to our emergency department (ED) with left-sided neck and shoulder pain, abdominal pain, and fever to 38.9^o^C. Seventy-two days prior he experienced fever, cough, and myalgias and tested positive for COVID-19 during this time. He described a complete recovery after three days. He did not require hospitalization, medications, or supplemental oxygen. COVID-19 test type and results were not available.

Eleven days prior to our ED evaluation and subsequent admission, he began to experience pleuritic chest pain. He was evaluated at a nearby ED after 48 hours of feeling unwell. He reported no additional symptoms. Upon presentation to that facility, his temperature was noted to be 37.5^o^C and his oxygen saturation was 99% on room air. His EKG was notable for a right bundle branch block, and normal sinus rhythm with a rate of 83. His troponin T of <0.01 ng/mL and D-dimer of 523 ng/mL (when age-adjusted) were both considered unremarkable according to institutional parameters. Bibasilar subsegmental atelectasis, left greater than right, was present on chest x-ray. His symptoms improved with ketorolac. A repeat troponin was unchanged, and he was considered low risk for both acute coronary syndrome and pulmonary embolism. He was discharged with non-steroidal anti-inflammatory drugs (NSAIDS). He was not tested for COVID-19 during this visit.

He first presented to our ED two days later with a fever of 38.3^o^C, persistent pleuritic chest pain, right posterior shoulder pain, and positional chest discomfort described as worse supine yet better sitting upright. His EKG showed a persistent right bundle branch block with no new ST-T changes. His chest x-ray was notable for nonspecific minimal interstitial prominences at the bases. His SARS-CoV-2 PCR Rapid test (Roche Diagnostics) was positive. His elevated D-Dimer of 756 ng/mL led to computed tomography (CT) chest angiogram notable for airspace disease and atelectatic change in both lower lobes. The findings were not suggestive of pneumonia or pulmonary emboli. There was no pericardial fluid or thickening. His troponin T (fifth generation, Roche Diagnostics) was considered unremarkable at 11 ng/L (reference normal ≤ 15 ng/L). His oxygen saturation during his ED visit remained 96% and above on room air. He was discharged with a possible new diagnosis of COVID-19 versus persistent positive testing and recommendations for continued supportive care.

**Figure 1:**
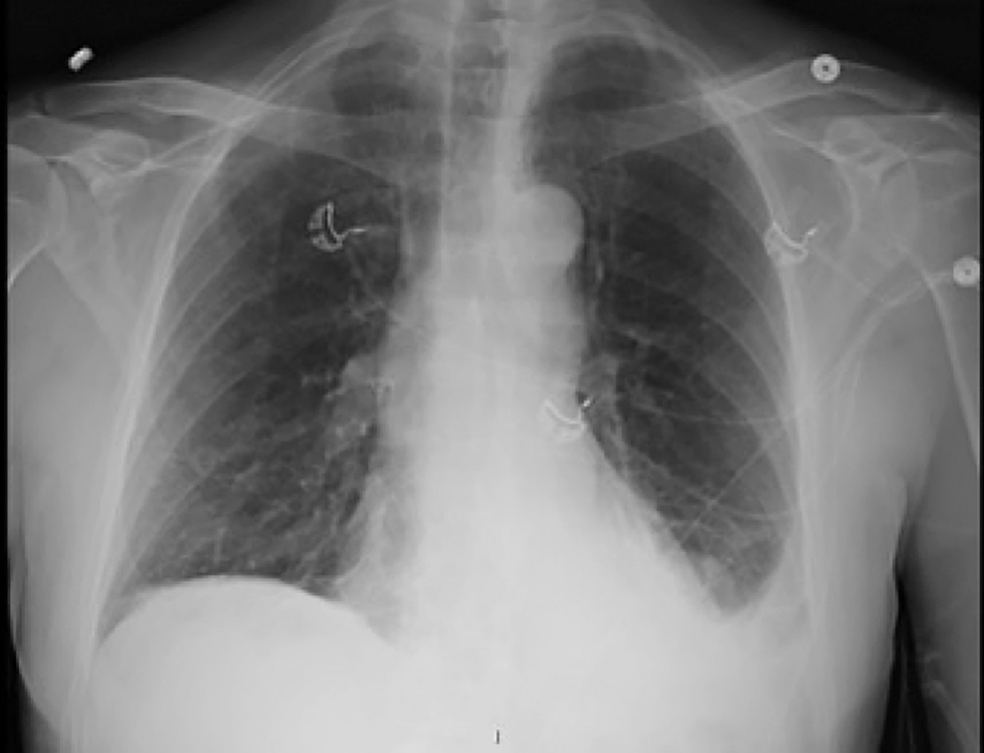
Chest x-ray with small left pleural effusion

**Figure 2:**
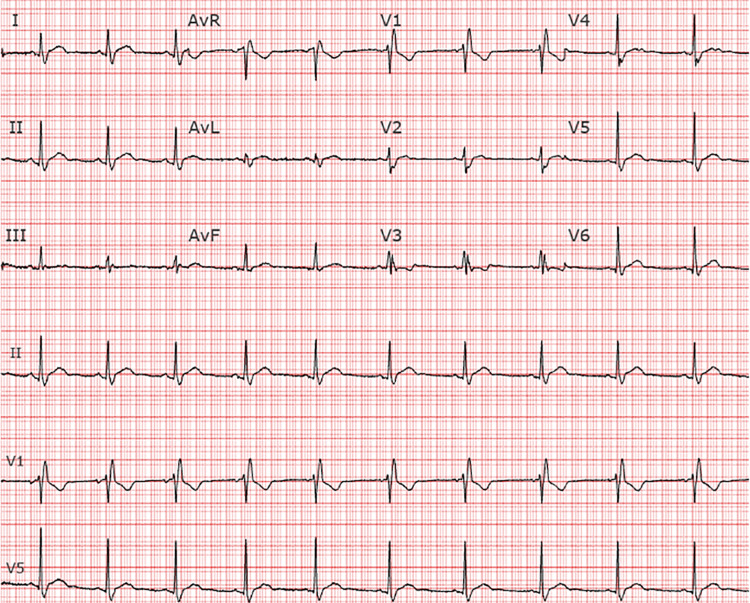
EKG with subtle PR depression and diffuse ST elevation

**Figure 3:**
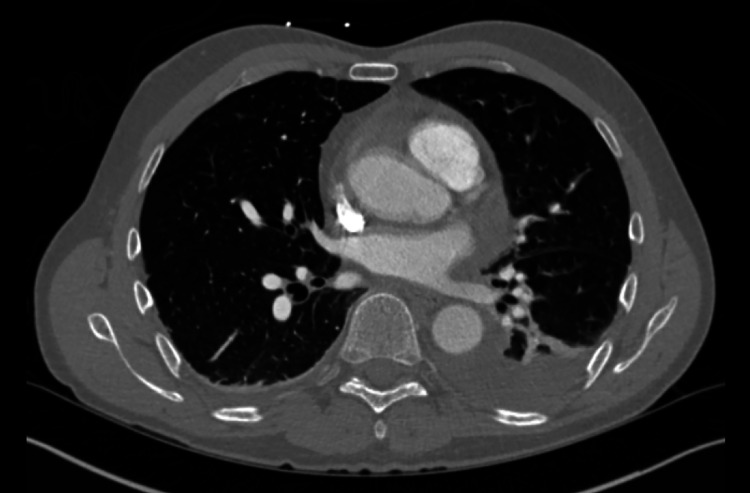
Computed tomography with small pericardial effusion, pericardial thickening, and left pleural effusion

**Figure 4:**
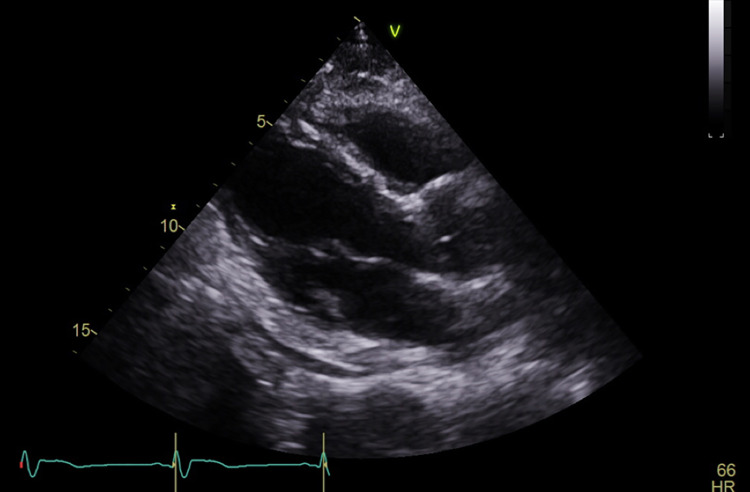
Echocardiogram with posterior pericardial effusion

Seven days later he returned to our ED with persistent symptoms. He continued to be febrile (39.2^o^C), and slightly hypoxic at times with peripheral oxygen saturations ranging between 91% and 96% on room air. His shoulder pain had worsened, and he was experiencing nausea, with both symptoms exacerbated by increases in his temperature at home. A repeat chest x-ray demonstrated a new small left-sided pleural effusion (Figure 1). His labs were notable for a white blood cell count of 8.1 x 10 (9)/L, troponin T, fifth generation of 15 ng/L, elevated NT-pro BNP 331 pg/mL (normal ≤ 89 pg/mL), elevated D-dimer 6,178 ng/mL, and lactate 1.3 mmol/L. His EKG showed a persistent right bundle branch block, and slight ST elevation in the inferior leads (Figure 2). Chest CT angiogram imaging revealed a new small pericardial effusion and pericardial wall thickening (Figure 3). No pulmonary emboli were noted. He was admitted for pericarditis due to possible reinfection versus sequelae of prior SARS-CoV-2 infection. His echocardiogram showed a small posterior pericardial effusion, normal regional wall motion and normal left ventricular size, as well as an ejection fraction of approximately 66% (Figure 4). Infectious disease consultation was obtained and the patient was diagnosed with possible extrapulmonary reinfection with COVID-19 pericarditis. Further inpatient workup revealed an elevated C-reactive protein 219.7 mg/L. On day 2 of admission SARS-CoV-2 antibodies were detected (Roche Elecsys Anti-SARS-CoV-2 Reagent assay). As he was stable without supplemental oxygen therapy, he did not meet our institutional threshold for corticosteroid therapy for acute COVID-19 infection. Cardiology consultation did, however, recommend prednisone and colchicine for the patient’s pericarditis. His symptoms improved shortly after prednisone initiation. He was additionally treated with remdesivir for presumptive ongoing COVID-19 versus extrapulmonary reinfection. He did not receive antibiotic therapy.

The patient was discharged on hospital day 7 after a five-day course of remdesivir. His CRP trended down to 33.2 mg/L on the day of discharge. His D-dimer peaked at 10,315 ng/mL on hospital day 6, decreasing to 7,447 ng/mL on his final hospital day. Ultrasound of the upper and lower extremities was negative. He was discharged home with 2.5 mg of apixaban twice daily for six weeks, based on institutional protocols for venous thromboembolism risk management in the setting of COVID-19 diagnosis, as well as a prednisone taper.

## Discussion

Pericarditis has been noted to be a rare presenting symptom of COVID-19, occurring at the time of or shortly after diagnosis [12-14]. Pericarditis and myocarditis presentations associated with positive COVID-19 PCR testing have ranged in reported acuity from mild symptoms to lethal tamponade. This case differs in its unusually late and subtle presentation. Our patient originally tested positive with complete symptomatic recovery, and developed a new fever, new pleural effusion and radiographic evidence of pericardial effusion over 70 days later. He did not have interim testing during his asymptomatic period. It is difficult to determine if this was a sequelae of prior infection three months earlier versus reinfection without viral sequencing data. It may be possible that the initial test was a false positive. Reinfection is rare but possible after initial infection, with early reports suggesting approximately five months of immunity after initial infection [15,16].

Often occurring after viral infections, pericarditis has been associated with coxsackievirus, herpesvirus, mumps, and HIV, and may present weeks after the initial illness. Other risk factors for pericarditis include prior myocardial infarction, trauma, end stage renal disease, cancer, specifically breast, lung, leukemia, non-Hodgkin’s and Hodgkin’s lymphoma, and medications including hydralazine and procainamide. This case is notable in its subtle presentation, EKG and echocardiogram findings, which required multiple evaluations prior to definitive diagnosis. Early diagnosis of pericarditis is essential as it is known complications include constrictive pericarditis and cardiac tamponade, with life-threatening presentations previously described in the setting of COVID-19 [12-14].

## Conclusions

It is important to remain vigilant for extrapulmonary manifestations of disease in patients with delayed nonspecific symptoms after COVID-19 infection. Regardless if it is from reinfection or latent COVID-19, pericarditis should be included on the differential for late-stage pleuritic symptoms, even without early evidence of EKG or CT imaging findings. Bedside ultrasound may facilitate early diagnosis and management of COVID-19 associated pericarditis which may improve outcomes.

## References

[1] Guan WJ, Ni ZY, Hu Y (2020). Clinical characteristics of coronavirus disease 2019 in China. N Engl J Med.

[2] Spinelli A, Pellino G (2020). COVID-19 pandemic: perspectives on an unfolding crisis. Br J Surg.

[3] Holshue ML, DeBolt C, Lindquist S (2020). First case of 2019 novel coronavirus in the United States. N Engl J Med.

[4] Baud D, Qi X, Nielsen-Saines K, Musso D, Pomar L, Favre G (2020). Real estimates of mortality following COVID-19 infection. Lancet Infect Dis.

[5] Depalo D (2020). True COVID-19 mortality rates from administrative data [PREPRINT]. J Popul Econ.

[6] Fraser E (2020). Long term respiratory complications of covid-19. BMJ.

[7] Katz JM, Libman RB, Wang JJ (2020). Cerebrovascular complications of COVID-19. Stroke.

[8] Terpos E, Ntanasis-Stathopoulos I, Elalamy I (2020). Hematological findings and complications of COVID-19. Am J Hematol.

[9] Shafi AMA, Shaikh SA, Shirke MM, Iddawela S, Harky A (2020). Cardiac manifestations in COVID-19 patients-A systematic review. J Card Surg.

[10] Kochi AN, Tagliari AP, Forleo GB, Fassini GM, Tondo C (2020). Cardiac and arrhythmic complications in patients with COVID-19. J Cardiovasc Electrophysiol.

[11] Rubin R (2020). As their numbers grow, COVID-19 "Long Haulers" stump experts. JAMA.

[12] Dabbagh MF, Aurora L, D'Souza P, Weinmann AJ, Bhargava P, Basir MB (2020). Cardiac tamponade secondary to COVID-19. JACC Case Rep.

[13] Inciardi RM, Lupi L, Zaccone G (2020). Cardiac involvement in a patient with coronavirus disease 2019 (COVID-19). JAMA Cardiol.

[14] Asif T, Kassab K, Iskander F, Alyousef T (2020). Acute pericarditis and cardiac tamponade in a patient with COVID-19: a therapeutic challenge. Eur J Case Rep Intern Med.

[15] Selvaraj V, Herman K, Dapaah-Afriyie K (2020). Severe, symptomatic reinfection in a patient with COVID-19. RI Med J.

[16] Hall V, Foulkes S, Charlett A (2021). Do antibody positive healthcare workers have lower SARS-CoV-2 infection rates than antibody negative healthcare workers? Large multi-centre prospective cohort study (the SIREN study), England: June to November [PREPRINT]. MedRxiv.

